# Novel Biomarkers for Outcome After Allogeneic Hematopoietic Stem Cell Transplantation

**DOI:** 10.3389/fimmu.2020.01854

**Published:** 2020-08-18

**Authors:** Sophia Chen, Robert Zeiser

**Affiliations:** ^1^Department of Immunology, Memorial Sloan Kettering Cancer Center, Sloan Kettering Institute, New York, NY, United States; ^2^Department of Medicine I, Faculty of Medicine, Medical Center, University of Freiburg, Freiburg, Germany; ^3^German Cancer Consortium (DKTK), Partner Site Freiburg, Freiburg, Germany; ^4^Signalling Research Centres BIOSS and CIBSS – Centre for Integrative Biological Signalling Studies, University of Freiburg, Freiburg, Germany

**Keywords:** biomarker, GVHD, steroid-refractory graft-vs.-host disease, immune cells, relapse, minimal residual disease

## Abstract

Allogeneic hematopoietic stem cell transplantation (allo-HSCT) is a well-established curative treatment for various malignant hematological diseases. However, its clinical success is substantially limited by major complications including graft-vs.-host disease (GVHD) and relapse of the underlying disease. Although these complications are known to lead to significant morbidity and mortality, standardized pathways for risk stratification of patients undergoing allo-HSCT are lacking. Recent advances in the development of diagnostic and prognostic tools have allowed the identification of biomarkers in order to predict outcome after allo-HSCT. This review will provide a summary of clinically relevant biomarkers that have been studied to predict the development of acute GVHD, the responsiveness of affected patients to immunosuppressive treatment and the risk of non-relapse mortality. Furthermore, biomarkers associated with increased risk of relapse and subsequent mortality will be discussed.

## Introduction

Allogeneic hematopoietic stem cell transplantation (allo-HSCT) is the only curative treatment for a variety of malignant hematological diseases. A major complication after allo-HSCT consists of acute graft-vs.-host disease (aGVHD), which occurs when immunocompetent T cells of the allo-HSCT donor recognize antigens on recipient cells as foreign and attack recipient tissue, mainly the skin, gastrointestinal tract and liver (1), but as shown more recently, also the central nervous system (2). Several immunosuppressive agents are used for the treatment of aGVHD (3). While aGVHD leads to significant morbidity and mortality, donor T cell effector functions are necessary for the elimination of remaining malignant cells after allo-HSCT. This phenomenon, termed graft-vs.-leukemia (GVL) effect, is crucial for reducing the risk of relapse of the underlying disease, a complication occurring in a large portion of patients and causing substantially reduced survival after allo-HSCT (4, 5). In order to improve outcome after allo-HSCT, it would be desirable to predict which patients are at a high risk to develop aGVHD, how they respond to corticosteroids and what their risk of non-relapse mortality (NRM) as well as relapse is. To address these questions, multiple candidate biomarkers have been determined and correlated with clinical outcome, with some having been validated in large patient cohorts.

## Biomarkers for Acute Graft-vs.-Host Disease and Non-relapse Mortality

Even when patients are cured of their underlying disease after allo-HSCT, their life expectancy remains inferior to that of age-matched general population due to NRM (6). Major risk factors of NRM include acute and chronic GVHD, infections, organ failure and second cancers (7). This review will focus on candidate and validated biomarkers that have been investigated in transplanted patients in order to predict the risk of aGVHD and the response to immunosuppressive therapy (Table 1).

**Table 1:** Candidate and validated biomarkers for aGVHD (alphabetical order).

**Biomarker name**	**Type of molecule (physiological function) - Association direction**	**Diagnostic significance**	**Prognostic significance**	**Predictive significance**	**Specimen analyzed**	**Number of patients analyzed**	**References**
Albumin	Protein (transport and oncotic pressure) - Decreased	ND	Grade III–IV aGVHD and increased 6-month NRM in patients undergoing reduced-intensity conditioning allo-HSCT	ND	Serum	401	(8)
Alpha-1-antitrypsin	Protein (protease inhibitor) - Increased	Stage II-III gastrointestinal aGVHD (vs. non-aGVHD diarrhea and aGVHD of other organs)	NS for 6-month survival	Steroid resistance of gastrointestinal aGVHD and lower cumulative incidence of complete response to steroids at 4 months	Feces	72	(9)
Angiopoietin-2	Protein (endothelial cell death and vessel regression) - Increased	ND	Increased NRM	Steroid resistance of aGVHD	Serum	48	(10)
α4β7 integrin	Protein (surface receptor, T cell homing into gut-associated lymphoid tissues) - Increased	ND	Occurrence of intestinal aGVHD	ND	Lymphocytes from PB (naïve and memory T cells)	59	(11)
B cell-activating factor	Protein (B cell activation) - Increased	ND	Occurrence of aGVHD	ND	Serum	Training cohort: 78, validation cohort: 37	(12)
Calprotectin	Protein (antimicrobial peptide) - Increased	NS	Decreased 6-month survival	Steroid resistance of intestinal aGVHD and lower cumulative incidence of complete response to steroids at 4 months	Feces	72	(9)
		Gastrointestinal aGVHD (vs. aGVHD of other organs and gastrointestinal infection)	ND	ND	Feces	68	(13)
CCL8	Protein (chemotaxis signal for various immune cells) - Increased	Grade I–IV aGVHD (vs. no aGVHD)	ND	ND	Serum	14	(14)
CD8, soluble	Protein (co-receptor for class I major histocompatibility complex T cell receptor) - Increased	ND	Grade III–IV aGVHD by day 60	ND	Plasma	62	(15)
CD30	Protein (TNFR superfamily member, proliferation of activated T cells) - Increased	ND	Grade III–IV aGVHD	ND	Plasma	30	(16)
		Grade I-IV aGVHD (vs. no aGVHD)	ND	ND	Plasma, lymphocytes from PB (CD8^+^ T cells)	53	(17)
CD31	Protein (endothelial cell marker) - Increased	ND	Grade III–IV aGVHD	ND	Intestinal biopsies (CD31^+^ cells)	27	(18)
CXCL10	Protein (ligand of CXCR3 expressed on T cells) - Increased	Grade I–IV aGVHD (vs. no aGVHD)	Grade I–IV aGVHD by day 100	ND	Serum	34	(19)
		ND	Occurrence of aGVHD	ND	Serum	Training cohort: 78, validation cohort: 37	(12)
Cytokeratin-18, fragmented^*^	Protein (intermediate filament in cytoskeleton) - Increased	Hepatic and intestinal aGVHD (vs. non-complicated infectious enteritis)	NS for NRM	Steroid resistance of hepatic and/or intestinal aGVHD	Serum	55	(20)
		Intestinal aGVHD (vs. non-aGVHD diarrhea and asymptomatic patients)	NS for 1-year NRM	Unresponsiveness to treatment at day 28	Plasma	954 (3 centers)	(21)
		ND	Occurrence of gastrointestinal/liver aGVHD	ND	Plasma	38	(22)
Elafin^*^	Protein (elastase-specific protease inhibitor) - Increased	Skin aGVHD (vs. non-aGVHD rash)	Decreased 5-year survival	ND	Plasma, skin biopsies	Discovery cohort: 522, validation cohort: 492	(23)
		NS for skin aGVHD (vs. drug hypersensitivity rash)	Decreased 2-year survival	ND	Skin biopsies	40	(24)
Glycero-phospholipid metabolites	Lipids (components of cell membranes) - Altered	ND	5-biomarker panel with altered glycerophospholipid metabolites at day 15 is associated with occurrence of aGVHD and reduced overall survival	ND	Plasma, RNA from PB	Discovery cohort: 57, validation cohort: 50	(25)
Hepatocyte growth factor^*^	Protein (liver regeneration after damage) - Increased	Grade I–IV aGVHD (vs. no aGVHD and healthy controls)	ND	ND	Serum	38	(26)
		Intestinal aGVHD (vs. non-aGVHD diarrhea and asymptomatic patients)	Increased 1-year NRM	Unresponsiveness to treatment at day 28	Plasma/serum	954 (3 centers)	(21)
IL-2Rα (CD25), soluble	Protein (α-chain cleaved from IL-2 receptor through extracellular proteolysis) - Increased	ND	Occurrence of aGVHD	ND	Serum	67	(27)
		ND	Grade III–IV aGVHD by day 60	ND	Plasma	62	(15)
		Grade I–IV aGVHD (vs. no aGVHD)	Occurrence of aGVHD	ND	Serum	13	(28)
		Grade II-IV aGVHD (vs. grade 0-I aGHVD)	ND	ND	Serum	18	(29)
		Skin-only and skin/visceral aGVHD (vs. visceral-only aGVHD)	ND	Lower incidence of complete responses to treatment at 4 weeks	Plasma	Discovery cohort: 42, training cohort: 282, validation cohort: 142	(30)
IL-2Rα/ TNFR1/ IL-8/ HGF^*^	Proteins - Increased	The 4-biomarker panel confirms the diagnosis of aGVHD	The 4-biomarker panel predicts higher NRM and lower overall survival at 2.5 years independent of GVHD severity	NS for responses to treatment at 4 weeks	Plasma	Discovery cohort: 42, training cohort: 282, validation cohort: 142	(30)
IL-6^*^	Protein (pro-inflammatory cytokine, activation of T cells, promotion of Th17 differentiation) - Increased	ND	Grade II–IV aGVHD	ND	Plasma	147	(31)
		ND	Grade III–IV aGVHD and increased 1-year NRM	ND	Plasma	First cohort: 74, second cohort: 76, landmark cohort: 167	(32)
IL-7	Protein (B and T cell development) - Increased	ND	Grade II–IV aGVHD	ND	Plasma	40	(33)
IL-10	Protein (anti-inflammatory cytokine, suppression of macrophage function, inhibition of Th1 cytokine production) - Increased	Grade II–IV aGVHD	ND	ND	Serum	34	(34)
		Grade I–IV aGVHD (vs. no aGVHD)	Increased NRM	ND	Serum	13	(28)
IL-12	Protein (induction of Th1 polarization) - Increased	ND	Grade II–IV aGVHD after reduced-intensity conditioning allo-HSCT	ND	Plasma	113	(35)
IL-15	Protein (common gamma chain cytokine, survival and proliferation of T cells) - Increased	ND	Grade III–IV aGVHD	ND	Plasma	13	(36)
IL-18	Protein (pro-inflammatory cytokine, promotion of Th1 induction; but also tissue-protective roles) - Increased	Grade II–III aGVHD	Occurrence of aGVHD	ND	Serum	67	(27)
		Grade I–IV aGVHD	ND	ND	Serum	37	(37)
miR-29a	microRNA - Increased	Grade I–IV aGVHD	Occurrence of aGVHD	ND	Serum	19, validation cohort 1: 60, validation cohort 2: 54	(38)
miR-146a	microRNA (anti-inflammatory) - Decreased	ND	Simultaneous low levels of both miR-146a and miR-155 at day 28 are associated with higher incidence of subsequent aGVHD	ND	Serum	54	(39)
		ND	The miR-146a polymorphism rs2910164 in the allo-HSCT donor or the recipient is connected to higher rates of grade III and IV aGVHD	ND	DNA from PB	286	(40)
					DNA from PB	289	(41)
miR-155	microRNA (pro-inflammatory) - Increased/Decreased	Grade I–IV aGVHD	ND	ND	Serum	64	(42)
		ND	Simultaneous low levels of both miR-146a and miR-155 at day 28 are associated with higher incidence of subsequent aGVHD	ND	Serum	54	(39)
		Intestinal aGVHD	ND	ND	Intestinal biopsies	8	(43)
miR-586	microRNA (pro-inflammatory) - Increased	aGVHD (and infection) (vs. time point before aGVHD)	Occurrence of aGVHD	ND	Plasma	52	(44)
miR-26b/ miR-374a	microRNAs - Decreased	ND	Occurrence of aGVHD	ND	Plasma	38, confirmation cohort: 54	(45)
miR-28-5p/ miR-489/ miR-671-3p	microRNAs - Decreased/Increased	The panel including miR-28-5p (decreased), miR-489 and miR-671-3p (increased) confirms aGVHD diagnosis	ND	ND	Plasma	38, confirmation cohort: 54	(45)
miR-194/ miR-518f	microRNAs - Increased	ND	Occurrence of aGVHD	ND	Plasma	24	(46)
REG3α^*^	Protein (antibacterial properties) - Increased	Intestinal aGVHD (vs. non-aGVHD diarrhea and asymptomatic patients)	Increased 1-year NRM	Unresponsiveness to treatment at day 28	Serum	954 (3 centers)	(21)
		Gastrointestinal GVHD (vs. no aGVHD and non-GVHD enteritis)	Increased 1-year NRM, decreased 1-year survival	Unresponsiveness to treatment at 4 weeks	Plasma	Discovery cohort: 20, validation cohorts: 871, 143	(47)
Stearic acid/palmitic acid ratio	Fatty acid - Decreased	ND	Low stearic acid/palmitic acid ratio on day 7 post-transplant is associated with grade II-IV aGVHD	ND	Serum	114	(48)
ST2^*^	Protein (IL-33 receptor) - Increased	ND	Increased 6-month NRM	Unresponsiveness to treatment by day 28	Plasma	Discovery cohort: 20, response-to-treatment cohort: 381, early stratification cohorts: 673, 75	(49)
		Grade I–IV aGVHD (cohort 2) and transplant-associated thrombotic microangiopathy (cohorts 2 and 3)	Increased 6-month NRM	ND	Plasma	3 cohorts: 95, 110, 107	(50)
		Grade I–IV aGVHD	ND	ND	Lymphocytes from PB (CD4^+^ T cells)	22	(51)
ST2/REG3α^*^	Proteins - Increased	ND	The 2-biomarker panel on day 7 after allo-HSCT identifies patients at high risk of GVHD-related mortality and 6-month NRM	ND	Plasma	Training cohort: 620, test cohort: 309, validation cohort: 358	(52)
		ND	The 2-biomarker panel measured 1 week after initiation of GVHD treatment predicts 1-year NRM and overall survival	The 2-biomarker panel measured 1 week after initiation of GVHD treatment identifies treatment unresponsiveness at week 4	Serum	Test cohort: 236, validation cohort: 142, 129	(53)
ST2/ REG3α/ TNFR1^*^	Proteins - Increased	ND	The combination of the three markers at the onset of GVHD symptoms predicts 6-month NRM	The combination of the three markers at the onset of GVHD symptoms predicts therapy unresponsiveness by day 28	Plasma	Training cohort: 328, test cohort: 164, validation cohort: 300	(54)
ST2/ TIM-3^*^	Proteins - Increased	NS	Increased NRM and decreased overall survival at 2 years	ND	Serum	211	(55)
TGF-β	Protein (pro- and anti-inflammatory function depending on the tissue context) - Decreased	ND	Occurrence of aGVHD	ND	Serum	13	(28)
		ND	Grade II-IV aGVHD	ND	Plasma	147	(31)
		ND	Grade II-IV aGVHD	ND	Serum	30	(56)
Thrombomodulin, soluble	Protein (inhibition of mitochondrial apoptosis of endothelial cells) - Increased	ND	Increased NRM	Increase of levels in patients with steroid-refractory aGVHD after escalation of therapeutic immunosuppression	Serum	48	(10)
TIM-3^*^	Protein (shredded version of a receptor causing negative regulation of T cell activation) - Increased	ND	Grade III–IV aGVHD	ND	Plasma	First cohort: 74, second cohort: 76, landmark cohort: 167	(32)
		Mid-gut aGVHD (vs. upper-gut aGVHD, no GVHD and normal controls)	Grade II–IV aGVHD	ND	Plasma, lymphocytes from PB (CD8^+^ T cells)	Discovery cohort: 20, validation cohorts: 127, 22	(57)
TNF-α	Protein (pro-inflammatory cytokine) - Increased	ND	Grade II–IV aGVHD and other transplant-related complications	ND	Serum	52	(58)
TNFR1	Protein (receptor for TNF) - Increased	ND	Increase of ≥ 2.5x on day 7 vs. pre-transplant baseline level is associated with grade II-IV aGVHD, higher transplant-related mortality and lower overall survival at 1 year	ND	Plasma	438	(59)
		ND	Grade III–IV aGVHD by day 60	ND	Plasma	62	(15)
Vascular endothelial-derived growth factor (VEGF)	Protein (promotion of angiogenesis) - Decreased	ND	High angiopoietin-2/VEGF ratio is associated with increased NRM	Decrease of VEGF levels in patients with steroid-refractory aGVHD after escalation of therapeutic immunosuppression	Serum	48	(10)

* *Validated biomarkers that underwent the steps of identification, verification and qualification according to the NIH consensus on biomarker criteria*.

*ND, not determined; NS, not significant*.

A **biomarker** is defined as a characteristic that is objectively measured and evaluated as an indicator of normal biological processes, pathogenic processes or pharmacologic responses to a therapeutic intervention (60). The *Biomarker Working Group of the National Institutes of Health (NIH) Consensus Development Project on Criteria for Clinical Trials in Chronic GVHD* as well as the *North-American and European Consortium* distinguished four categories of GVHD biomarkers (61, 62): (1) **diagnostic** biomarkers, which identify GVHD patients at the onset of clinical disease, (2) **prognostic** biomarkers, which categorize patients by degree of risk for GVHD occurrence, progression or resolution before the onset of clinical disease, (3) **predictive** biomarkers, which categorize patients by their likelihood of response or outcome to a particular treatment before initiation of the treatment, and (4) **response-to-treatment** biomarkers, which monitor patients' response to GVHD treatment after initiation of therapy and which can substitute for a clinical efficacy endpoint.

Before being considered for standard clinical use, the development of biomarkers has to undergo a multi-step process consisting of (61): (1) **identification** of potential biomarker candidates in a small experiment of well-matched cases and controls selected from the populations in which the biomarker is intended for use, (2) **verification** by confirming the analytical validity and practicality of the test in an independent patient cohort, and (3) **qualification** by testing the impact on patient outcomes.

### Immune Cell-Derived Biomarkers

Early approaches to identify biomarkers for aGVHD mainly focused on the detection of inflammatory cytokines involved in the pathogenesis of the disorder. Increased levels of interleukin (**IL**)**-12** and **IL-18**, two cytokines known to promote T cell differentiation into T helper (Th) 1 cells with subsequent interferon-γ production, have been shown to correlate with severity of aGVHD (27, 35, 37). High levels of the key pro-inflammatory cytokine tumor necrosis factor (**TNF**)**-α**, mainly produced by macrophages, as well as elevated serum levels of its receptor **TNFR1** were also found to be associated with severe aGVHD (15, 58, 59). Studies on another pro-inflammatory cytokine, **IL-6**, validated that increased levels at the time period before or at the onset of GVHD symptoms predicted development of severe GVHD (31, 32). Several studies described an association between levels of soluble IL-2 receptor α (**IL-2Rα**) and the occurrence of aGVHD (15, 27–29). Furthermore, IL-2Rα levels at GVHD onset were associated with complete responses to treatment at 4 weeks (30). B cell-activating factor (**BAFF**) as an indicator of B cell activation was also found to be increased pre-transplant and on day 14 in aGVHD patients (12).

**Figure 1:**
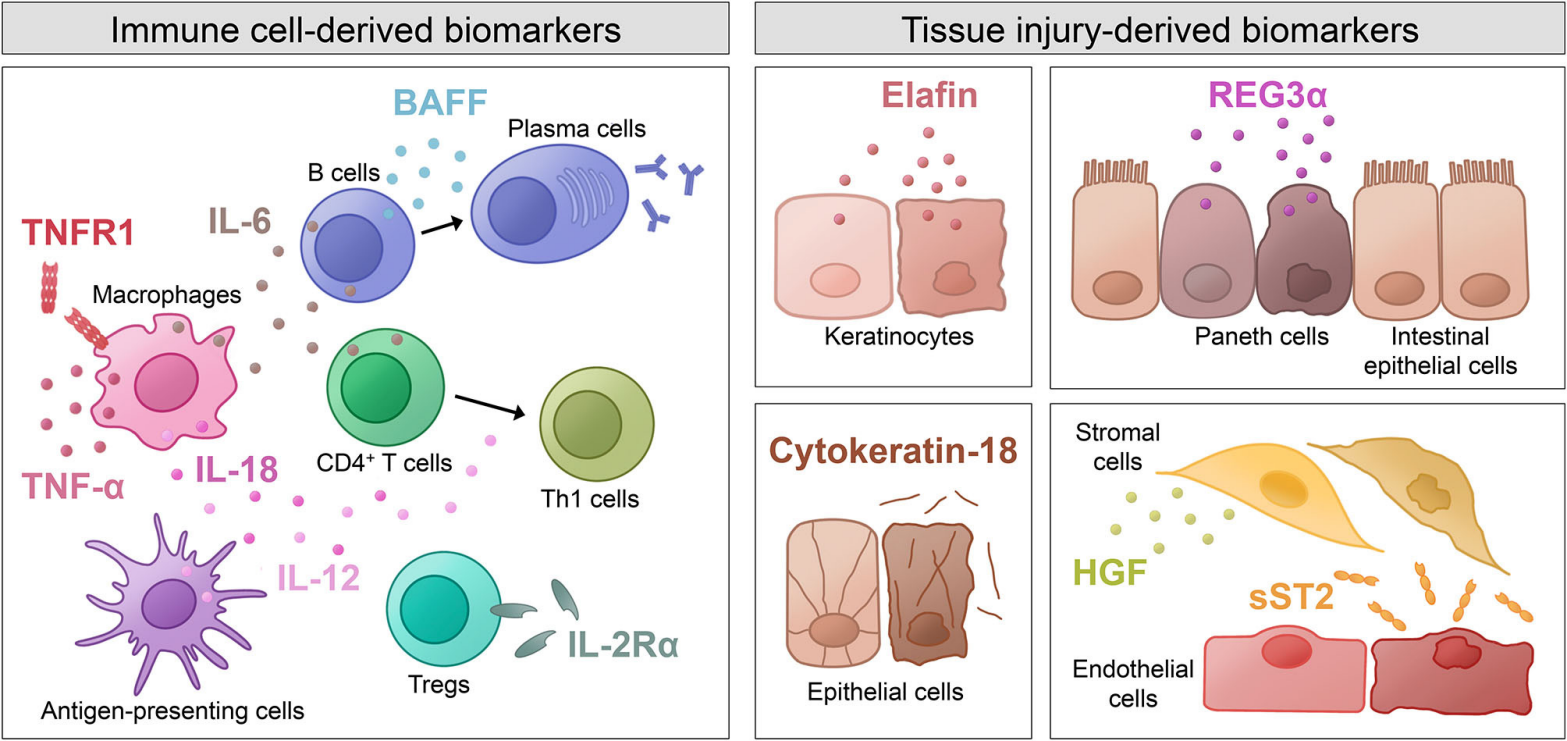
Shown are immune cell-derived molecules and tissue injury-derived molecules as well as the cells that they originate from. The molecules have various physiological functions and were described as biomarkers for acute GVHD. BAFF, B cell-activating factor; HGF, hepatocyte growth factor; IL, interleukin; REG3α, regenerating islet-derived protein 3α; sST2, soluble isoform of suppression of tumorigenicity 2; Th1 cells, T helper 1 cells; TNF-α, tumor necrosis factor α; TNFR1, tumor necrosis factor receptor 1; Tregs, regulatory T cells.

Not only have increased levels of various pro-inflammatory cytokines (depicted in Figure 1) been identified as potential biomarkers for aGVHD, but also cytokines with anti-inflammatory effects and their dysregulation have been investigated. Decreased levels of transforming growth factor β (**TGF-β**), which is involved in the generation of regulatory T cells (Tregs) and inhibition of lymphocyte activation, have been associated with GVHD incidence and severity (28, 31, 56). Interestingly, **IL-10**, which is known to suppress macrophage functions and inhibit expression of Th1 cytokines, was demonstrated to be increased in aGVHD patients (28, 34). The authors hypothesize that high levels of IL-10 during GVHD are produced in response to the existing inflammation in order to inhibit further production of pro-inflammatory cytokines.

Other molecules found in the plasma that are related to immune cell activation and that were investigated as potential biomarkers in aGVHD include chemokines, such as **CXCL10** and **CXCL11** as mediators of leukocyte chemotaxis (12), the soluble extracellular domain of T cell immunoglobulin and mucin domain 3 (**TIM-3**) (32, 57) and **α4β7 integrin**, a surface molecule involved in lymphocyte trafficking to intestinal lymphoid tissue (11).

### Tissue Injury-Derived Biomarkers

Novel advances in proteomic analyses have allowed screening of large numbers of patient samples and identification of novel biomarker candidates. Some of these potential biomarkers are not directly involved in the pathogenesis of aGVHD, but rather indicate end-organ tissue injury caused by the inflammatory processes in aGVHD (depicted in Figure 1). Since certain molecules are released from particular cell types, some biomarkers have diagnostic value for specific GVHD target organs. For instance, **elafin**, an elastase-specific protease inhibitor, was identified as a diagnostic and prognostic biomarker for skin GVHD, which is associated with higher incidence and lower overall survival (23, 24). Regenerating islet-derived protein 3α (**REG3α**), a C-type lectin secreted by Paneth cells, was validated as a prognostic marker for aGVHD of the intestinal tract (47). When epithelial cell death occurs, the intermediate filament **cytokeratin-18** is cleaved, and the fragments released into the serum were found to be elevated in patients with intestinal and liver GVHD (20–22). Hepatocyte growth factor (**HGF**), a molecule involved in tissue repair, was shown to be elevated in liver GVHD patients, probably due to increased release from the target organ as a physiologic response to GVHD tissue damage (21, 26). A marker that indicates tissue damage especially in endothelial and stromal cells is the soluble form of suppression of tumorigenicity 2 (**ST2**). ST2 is a member of the IL-1 receptor family with a transmembrane isoform and a soluble (sST2) isoform. Latter acts as a decoy receptor for IL-33 and was shown to correlate with the risk of therapy-resistant aGVHD and 6-month NRM (49).

### Plasma Biomarker Panels

A large number of molecules in the plasma have been identified as potential biomarkers, but changes observed in single candidates mostly lacked sufficient specificity and sensitivity to be introduced into routine clinical use. A first 4-biomarker panel consisting of **IL-2Rα, TNFR1, IL-8**, and **HGF** was validated for confirmation of aGVHD diagnosis and prediction of survival independent of GVHD severity (30). A combination algorithm using the concentrations of **ST2, REG3α**, and **TNFR1** measured at the onset of aGVHD symptoms was developed to assess therapy responsiveness within 28 days and the probability of 6-month NRM (54). The combination of **ST2** and **REG3α** measured 7 days after allo-HSCT was shown to be connected to increased aGVHD-related death risk (52). The same algorithm using high levels of ST2 and REG3α applied 1 week after the initiation of GVHD treatment was able to identify treatment unresponsiveness at week 4 (53).

### Metabolic Biomarkers

Given that the type of saturated fatty acid present in the diet can significantly affect lymphocyte functions (63), an untargeted metabolomics study demonstrated that patients with lower **serum stearic acid**/**palmitic acid** ratios on day 7 after transplantation were more likely to develop aGVHD, while no differences in NRM were observed (48). Another study reported significant variation in microbiota-derived metabolites at the onset of aGVHD, especially in **aryl hydrocarbon receptor ligands**, **bile acids** and **plasmalogens** (64). A recent integrated metabolomics and transcriptomics study uncovered an altered glycerophospholipid (**GPL**) **metabolism** signature of aGVHD, which was used to develop a biomarker panel with prognostic value using five GPL metabolites (25).

### MicroRNAs as Biomarkers

Besides soluble factors in the blood of the GVHD patients, microRNAs (miRs), which determine the transcription of multiple target genes, were evaluated after allo-HSCT [reviewed in (1)]. MiRs are potent regulators of multiple pro-inflammatory target genes and readily measurable in patient serum. Multiple miRs in the serum were strongly connected to aGVHD risk (46, 65), in particular **miR-155** and **miR-146a** (39, 42). MiRs, such as miR-155, can also be found in intestinal biopsies of patients with aGVHD (43). Several miRs were studied in mouse models of GVHD and were shown to promote or inhibit GVHD, including miR-155 (43, 66), miR-146a (40, 41), and miR-100 (18). MiR-155 was found to be essential for CXCR4-dependent donor T cell migration during GVHD (43) and NLRP3 inflammasome activation in dendritic cells (66). The miR-146a polymorphism rs2910164 in either the allo-HSCT donor or recipient was connected to higher rates of grade III and IV aGVHD (40, 41).

### Microbiome-Associated Changes as Biomarkers

Major shifts in the composition of the intestinal flora have been observed during allo-HSCT as well as GVHD (67). Different studies showed that **loss of intestinal microbiota diversity** and **predominance of a single bacterial genus**, e.g., *Enterococcus*, were associated with occurrence of intestinal GVHD as well as overall mortality after engraftment (67, 68). On the other hand, harboring increased amounts of bacteria belonging to the genus *Blautia* was associated with reduced GVHD mortality in two independent cohorts (69). Another study identified increases in Lactobacillales and decreases in Clostridiales at GVHD onset (70). These shifts in species abundance and measures of diversity [reviewed in (71)] could potentially serve as biomarkers for outcome after allo-HSCT.

## Biomarkers for Relapse

Relapse of the underlying disease is the main cause of death in the first years after allo-HSCT (72, 73). Leukemia cells use various mechanisms to escape the allogeneic immune system, such as loss of human leukocyte antigen (HLA) molecules (74), downregulation of HLA expression (75), upregulation of immune checkpoint ligands (76) and others [reviewed in (77)]. A summary of various biomarkers that have been evaluated for prediction of relapse can be found in Table 2.

**Table 2:** Biomarkers for relapse (alphabetical order).

**Biomarker name**	**Main message on association with relapse**	**Specimen analyzed**	**Number of patients analyzed**	**References**
ALL MRD	MRD positivity at day 60 after allo-HSCT or beyond is highly predictive for subsequent relapse.	BM	113	(78)
BCR-ABL	Relative risk of relapse is significantly higher for patients with a detectable BCR/ABL transcript following allo-HSCT.	BM	30	(79)
CBFB-MYH11	CBFB-MYH11 transcript levels that decreased by <3 logs compared with pre-treatment baseline levels at 1, 2 and 3 months after allo-HSCT are predictive for relapse.	BM	53	(80)
Chimerism	Relapse is more frequent in patients with MC than in patients with CC.	PB, BM	101	(81)
	Patients with MC on day 90 after allo-HSCT are at higher risk of relapse and have lower disease-free survival and overall survival when compared with patients with CC.	BM	69	(82)
	The cumulative incidence of relapse is significantly higher in ALL patients with increasing MC compared with those with CC.	PB, BM	101	(83)
	Decrease of CD34^+^-specific donor chimerism to <80% can predict relapse.	CD34^+^ cells from PB	14	(84)
	T lymphocyte chimerism ≤ 85% at days 90 and 120 after allo-HSCT predicts relapse for AML/MDS patients who were in first/second complete remission at transplantation.	T cells from PB	378	(85)
DNMT3A	Patients with persistent ctDNA^+^ status of *DNMT3A* and other founder mutations either at 1 month or 3 months post-allo-HSCT have a higher risk of relapse and death.	ctDNA from PB, BM	51	(86)
FLT3-ITD	Reduction in *FLT3-ITD* mutation burden after gilteritinib treatment in patients with relapsed or refractory AML is associated with longer median overall survival.	BM	80	(87)
IL-15	Lower peak levels of IL-15 on day 14 after transplantation are associated with subsequent occurrence of malignancy relapse.	Plasma	40	(33)
MLL	MLL positivity is associated with a higher rate of relapse, lower leukemia-free survival and lower overall survival.	BM	40	(88)
NPM1	Persistent *NPM1* mutation-based MRD after allo-HSCT is associated with increased incidence of relapse.	BM	53	(89)
		BM	174	(90)
		BM	59	(91)
RUNX1-RUNX1T1	RUNX1/RUNX1T1-based MRD status during the first 3 months after allo-HSCT is highly predictive for post-transplant relapse for *t*(8;21) patients.	BM	92	(92)
		BM	208	(93)
Stearic acid/palmitic acid ratio	High stearic acid/palmitic acid ratio on day 7 after transplantation is associated with increased risk of relapse.	Serum	114	(48)
WT1	Continuous increase of PB-WT1 transcripts and high levels of pre-transplant BM-WT1 transcripts at 3 months post-allo-HSCT are associated with increased risk of relapse.	PB	59	(94)
		BM	425	(95)

### Measurable Residual Disease

Measurable residual disease (MRD, also referred to as minimal residual disease) can be used to identify remaining leukemic cells that are below the limit of detection of morphological assessment (96). MRD monitoring can thus help to identify patients with increased risk of relapse after allo-HSCT. However, not all patients with MRD positivity will relapse clinically, and some patients will relapse despite negative MRD results. The following paragraphs will focus on MRD detection in acute myeloid leukemia (AML) and acute lymphoblastic leukemia (ALL), which, taken together, account for a large portion of indications for allo-HSCT (97).

Given the molecular diversity of acute leukemia, different methods are applied for MRD detection. Multiparameter flow cytometry and real-time quantitative polymerase chain reaction (PCR) are widely used, while newer technologies are emerging, e.g., droplet digital PCR (ddPCR) and next-generation sequencing (NGS) (98).

Overexpression of Wilms tumor 1 (***WT1***) is found in most AML patients and can be measured in peripheral blood (PB) or bone marrow (BM) (99, 100). Patients who displayed increased WT1 transcripts in the PB after allo-HSCT or who failed to clear their high levels of pre-transplant WT1 transcripts in the BM at 3 months post-allo-HSCT were shown to be at increased risk of relapse (94, 95). Mutation in nucleophosmin 1 (***NPM1***) is present in around one-third of adult AML patients (101). Several studies showed an association between persistent *NPM1* mutation-based MRD after allo-HSCT and increased incidence of relapse (89–91). Core binding factor (CBF) AML is characterized by the presence of the chromosomal rearrangements *t*(8;21) and inv(16), causing production of the fusion transcripts RUNX1/RUNX1T1 and CBFB-MYH11, respectively (102). **RUNX1/RUNX1T1**-based MRD status in *t*(8;21) AML patients during the first 3 months after allo-HSCT was found to be highly predictive for post-transplant relapse (92). Similarly, **CBFB-MYH11** transcript levels that decreased by <3 logs compared with pre-treatment baseline levels at 1, 2, and 3 months after allo-HSCT were demonstrated to be predictive for relapse (80). Interestingly, low levels of CBF fusion transcripts were observed to persist in long-term transplant survivors (103). The mixed leukemia lineage (***MLL***) gene (also termed *KMT2A)*, is frequently disrupted in AML by different chromosomal rearrangements involving other partner chromosomes (104). MLL positivity was shown to be associated with a higher rate of relapse, lower leukemia-free survival and lower overall survival (88). The detection of driver mutations associated with clonal hematopoiesis of indeterminate potential (CHIP), such as mutations in ***DNMT3A***, ***TET2*, **and ***ASXL***, is complex because these mutations might be derived from the allo-HSCT donor (105). Some studies indicate that residual allelic burdens associated with CHIP were not suitable for MRD testing in remission to predict relapse rate (106, 107). However, in a study utilizing personalized ddPCR, patients with persistent ctDNA^+^ status of *DNMT3A* and other driver mutations either at 1 or 3 months post-allo-HSCT had a significantly higher risk of relapse and death compared with those with negative status (86). Additionally, increasing ctDNA levels between 1 and 3 months post-allo-HSCT was a precise predictor of relapse (86). Mutations in the fms-like tyrosine kinase 3 (*FLT3*) gene producing internal tandem duplications (**FLT3-ITD**) are common in AML and are known to be associated with poor prognosis (108). A novel NGS-based MRD assay detecting FLT3-ITD showed that reduction in mutation burden after treatment with gilteritinib, a FLT3 inhibitor, in patients with relapsed or refractory AML (NCT02014558) was linked to longer median overall survival (87). Also, RAS mutations (**NRAS** and **KRAS**) can be detected after allo-HSCT, and a link of KRAS downstream signaling with NLRP3 inflammasome activation was recently reported (109), showing a potential pro-inflammatory activity of certain oncogenic mutations.

MRD monitoring in B- or T-lymphoid malignancies includes detection of a **leukemia-associated immunophenotype** (LAIP) by flow cytometry as well as detection of disease-specific **T cell receptor or immunoglobulin gene rearrangements** by PCR (110, 111). Several studies in the pediatric setting of ALL have shown that patients with detectable MRD after allo-HSCT were more likely to experience relapse (78, 112, 113). In adult patients with Philadelphia chromosome-positive ALL, MRD positivity in terms of detectable **BCR/ABL** transcript was found to be associated with increased risk of relapse (79).

### Chimerism

Studies on different hematological malignancies showed the relevance of chimerism and its kinetics for the prediction of relapse (110). For instance, the cumulative incidence of relapse was found to be significantly higher in patients with AML, myelodysplastic syndrome (MDS), chronic myeloid leukemia (CML) and ALL with increasing **mixed chimerism** (MC) than in those with complete chimerism (CC) (81–83). Lineage-specific chimerism analysis may increase the specificity in predicting relapse (114). A prospective study found that the decrease of CD34^+^-specific donor chimerism to <80% had 100% sensitivity and 86% accuracy in predicting relapse (84). T lymphocyte chimerism ≤ 85% at days 90 and 120 after allo-HSCT was shown to predict relapse for patients who were in first/second complete remission at transplantation (85).

### Plasma Biomarkers

Levels of **ST2** and **REG3α** were previously used to develop an algorithm that predicts the risk of severe GVHD and NRM. The authors used this same algorithm to show that low levels of ST2 and REG3α on day 28 after allo-HSCT in patients who had not developed GVHD were associated with higher risk of relapse than severe GVHD and NRM (115). This observation suggests that the patients who are at low risk of developing severe GVHD, but who remain at an increased risk of relapse, might benefit from early taper of prophylactic immunosuppression in order to enhance GVL effects. Low peak levels at day 14 of another candidate biomarker connected to aGVHD, **IL-15**, were shown to be associated with subsequent occurrence of malignancy relapse (33).

A recent study aimed to develop a plasma signature to identify GVL effects without GVHD by conducting plasma proteomics and systems biology analyses of patients in relapse after allo-HSCT who were treated with allogeneic donor lymphocyte infusions (116). A unique 61-protein signature was identified in patients with GVL without GVHD, of which 43 genes were further confirmed using single-cell RNA sequencing analysis in activated T cells. Novel markers, such as **RPL23**, **ILF2**, **CD58**, and **CRTAM**, were identified and will need further validation in other cohorts.

### Metabolic Biomarkers

An untargeted metabolomic study showed that in a patient cohort with AML, ALL and Non-Hodgkin lymphoma, a high ratio between **serum stearic acid** and **palmitic acid** on day 7 after transplantation was associated with increased risk of relapse, suggesting that the measurement of this ratio may improve risk stratification after allo-HSCT (48).

## Conclusion

Acute GVHD and relapse of the underlying disease form the two major complications after allo-HSCT, leading to significant morbidity and mortality. Recent advances in proteomic analyses allowed the identification of numerous candidate biomarkers for aGVHD. Of note, the discovery of these candidate biomarkers was mostly based on evaluation at a single center and only a limited number of studies met the criteria of verifying and qualifying these candidates as actual biomarkers according to the NIH consensus. Those and possibly other yet to be discovered biomarkers hold promise to better predict the risk of aGVHD and aGVHD-related mortality, which could lead to a more individualized GVHD prophylaxis approach. Monitoring of MRD and chimerism is the most commonly used tool to detect relapse after allo-HSCT. The ultimate significance of MRD monitoring, in particular, remains to be further investigated. MRD detection techniques are constantly improving. However, clinical trials will be necessary to define standardized pathways for MRD testing and MRD-directed therapy intervention in clinical practice.

## Author Contributions

SC and RZ wrote the manuscript together. All authors contributed to the article and approved the submitted version.

## Conflict of Interest

RZ received speaker fees from Novartis, Incyte, and Mallinckrodt. The remaining author declares that the research was conducted in the absence of any commercial or financial relationships that could be construed as a potential conflict of interest.
